# Association between glucose and lipid metabolism-related indicators and the occurrence and severity of pelvic organ prolapse in postmenopausal women

**DOI:** 10.3389/fmed.2026.1876723

**Published:** 2026-07-08

**Authors:** Dawei Cui, Mengjie Yang, Can Cui, Lin Zhang, Yingchang Dai, Shuli You, Xin Luo

**Affiliations:** 1 Department of Gynecology, Jinhua Municipal Central Hospital (Affiliated Jinhua Hospital, Zhejiang University School of Medicine), Jinhua, Zhejiang, China; 2 Depertment of Gynecology, Jinan University, Guangzhou, China; 3 Department of Gynecology, Women & Children’s Hospital of Hunan, Changsha, Hunan, China; 4 Department of Radiology, Jinhua Municipal Central Hospital (Affiliated Jinhua Hospital Zhejiang University School of Medicine), Jinhua, Zhejiang, China; 5 Department of Gynecology, Wucheng District People's Hospital, Jinhua, Zhejiang, China

**Keywords:** glucose metabolism, lipid metabolism, ordinal logistic regression, pelvic organ prolapse (POP), postmenopausal women, prediction model

## Abstract

**Background:**

Studies have shown that the occurrence of pelvic organ prolapse (POP) is associated with abnormalities in collagen, while glucose and lipid metabolism have also been proven to be related to collagen; however, their relationships with the risk and severity of POP remain unclear.

**Methods:**

This retrospective study included 280 postmenopausal women, among whom 118 were in the POP group and 162 were in the non-POP group. Univariate and multivariate logistic regression analyses were performed to identify factors associated with POP and to develop a prediction model. Restricted cubic spline (RCS) analysis was used to evaluate the linear or nonlinear association between the prediction model and the risk of POP. Multivariate Logistic regression and ordinal Logistic regression analyses were performed to assess the relationships between the prediction model and the risk and severity of POP, while adjusting for potential confounding factors such as age and body mass index (BMI). Stratified analysis was further conducted to determine the applicable population of the model.

**Results:**

FPG (OR = 1.028, 95%CI: 1.004–1.052, *p* = 0.023), HbA1c (OR = 1.073, 95%CI: 1.036–1.111, *p* < 0.001), TG (OR = 1.074, 95%CI: 1.003–1.149, *p* = 0.041), and LDL (OR = 1.069, 95%CI: 1.015–1.126, *p* = 0.012) were independent risk factors, whereas 25(OH)D was a protective factor. The prediction model demonstrated moderate discriminative ability (AUC = 0.721) and showed a linear or approximately linear positive correlation with the risk of POP, as well as a significant positive correlation with the severity of POP. These associations remained significant after adjusting for confounding factors. In addition, there was a significant interaction between the prediction model and age (OR = 1.048, 95%CI: 1.005–1.093, *p* = 0.028).

**Conclusion:**

The prediction model based on glucose and lipid metabolism constructed in this study can effectively assess the risk and severity of POP.

## 1 Introduction

Pelvic organ prolapse (POP) is a pelvic floor dysfunction disease caused by the weakening of pelvic floor muscles and fascial tissues (1, 2), commonly occurring in postpartum or perimenopausal women (3, 4). Studies have shown that the lifetime risk of POP in elderly women is estimated to be between 30 and 50%, while the prevalence of symptomatic prolapse ranges from 3 to 12% (5). POP often leads to urinary frequency, urgency, stress urinary incontinence (6), and is accompanied by significant psychological burden and limitations in social activities, severely reducing patients’ overall quality of life. It has become an important disease burden affecting the health of middle-aged and elderly women. Therefore, early identification of POP is necessary in order to enable intervention at an early stage of the disease and improve patient prognosis.

In postmenopausal women, a significant decline in estrogen levels can lead to physiological changes across multiple systems, mainly involving metabolism, the cardiovascular system, and the genitourinary system (7, 8). Among these, metabolic changes primarily include abnormalities in glucose metabolism and increased insulin resistance. Metabolic abnormalities may lead to various diseases, including POP. Compared with normal-weight women, those in the overweight and obese categories have a 36 and 47% increased risk of developing POP, respectively (9). In addition, metabolic syndrome (MS) and elevated triglyceride levels are associated with increased severity of POP, with MS and hypertriglyceridemia serving as significant predictors of POP-Q stage ≥ III (10). Most studies have also found lower vitamin D levels in women with POP (11). Although the relationship between metabolic abnormalities and POP has been partially clarified in women with gestational diabetes mellitus (GDM) (12), significant differences exist between GDM populations and postmenopausal women in terms of age structure, hormonal status, and physiological conditions. Therefore, findings from GDM-related studies cannot be directly extrapolated to postmenopausal women. Systematic evidence regarding the association between metabolic factors and POP in postmenopausal women remains limited.

Current studies have shown that the occurrence of POP is closely related to abnormalities in collagen metabolism in pelvic floor connective tissues (13). Meanwhile, previous research has also suggested that disorders of glucose metabolism and lipid metabolism may, to some extent, be involved in alterations in the structure and function of connective tissues (14, 15). Based on this, the present study aims to investigate the associations between glucose and lipid metabolism and both the presence of POP and anatomical POP-Q stage, which may help further elucidate the underlying metabolic-related mechanisms and provide new insights for POP risk assessment.

## 2 Methods

### 2.1 Study population

This study was a retrospective study that included women who visited the Department of Gynecology in our hospital from January 2021 to January 2024.

Inclusion criteria:

1 Postmenopausal women (natural menopause ≥12 months)
2 Complete laboratory examination data
3 Patients in the POP group met the clinical diagnostic criteria of the International Continence Society (ICS) (16)

Exclusion criteria:

1 Malignant tumors or severe systemic diseases
2 Use of glucocorticoids within 3 months prior to blood sample collection
3 Missing outcome data

### 2.2 Data collection

Demographic and clinical characteristics, including age, age at menopause, and body mass index (BMI), were collected through the hospital electronic medical record system. Laboratory indicators included fasting plasma glucose (FPG, mmol/L), glycated hemoglobin (HbA1c, %), homeostasis model assessment of insulin resistance (HOMA-IR), total cholesterol (TC, mmol/L), triglycerides (TG, mmol/L), high-density lipoprotein cholesterol (HDL-C, mmol/L), low-density lipoprotein cholesterol (LDL-C, mmol/L), C-reactive protein (CRP, mg/L), and 25-hydroxyvitamin D [25(OH)D, ng/mL].

### 2.3 Outcome measures

Patients were placed in the lithotomy position, and pelvic floor physical examinations were performed by experienced gynecologists. During maximal Valsalva maneuver, the descent of anatomical reference points of the anterior vaginal wall, posterior vaginal wall, and vaginal apex relative to the hymenal plane was measured, and staging was performed accordingly. The POP-Q system classifies pelvic organ prolapse into stages 0–IV: Stage 0 indicates no prolapse (all measurement points are above the hymen); Stage I indicates prolapse that does not reach the mid-vagina (the most distal portion of prolapse remains more than 1 cm above the hymen); Stage II indicates prolapse close to or reaching the vaginal opening (the most distal portion is within ±1 cm of the hymenal plane); Stage III indicates prolapse beyond the vaginal opening but not complete eversion (the most distal portion extends more than 1 cm beyond the hymen but does not reach the total vaginal length [TVL] − 2 cm); and Stage IV indicates complete eversion of pelvic organs (the most distal portion is ≥ [TVL − 2] cm or there is complete vaginal eversion).

### 2.4 Statistical analysis

This study was analyzed using R version 4.4.0 software. Continuous variables were first tested for normality. Variables with a normal distribution were expressed as mean ± standard deviation, and comparisons between groups were performed using the independent samples t-test; variables that did not follow a normal distribution were expressed as median (interquartile range) and analyzed using the Mann–Whitney U test. Categorical variables were expressed as counts (percentages), and comparisons between groups were conducted using the χ^2^ test or Fisher’s exact test, with results further adjusted by false discovery rate (FDR) correction. Biochemical indicators were treated as independent variables, and the presence of POP was used as the dependent variable for multivariate Logistic regression analysis and collinearity testing. A prediction model was constructed based on the regression coefficients of significant variables, and its discriminative ability was evaluated using the receiver operating characteristic (ROC) curve and the area under the curve (AUC). Internal calibration analysis of the prediction model was performed using the “rms” package with bootstrap resampling (1,000 iterations), and a calibration curve was plotted. Three multivariable logistic regression models were constructed to evaluate the robustness of the association between the prediction model and POP. Model 1 included only the prediction model and was used to assess its crude association with POP risk. Model 2 was further adjusted for age and BMI, both of which are well-established risk factors for POP and may also influence metabolic indicators. Model 3 was additionally adjusted for parity, history of hysterectomy, T2DM, hypertension, COPD, CKD, CAD, and osteoporosis as potential confounding factors, in order to determine whether the association between the prediction model and POP remained independent after controlling for demographic characteristics, medical history, and comorbidities. Similarly, three ordinal Logistic regression models were constructed, with the same independent variables as above and POP stage (Stage I–IV) as the dependent variable. Bootstrap resampling (1,000 iterations) was performed to assess the robustness of the models. Restricted cubic spline (RCS) analysis was used to evaluate the dose–response relationship and its linear or nonlinear trend between the prediction model and the risk of POP. Interaction analysis was performed to assess the stability and applicability of the prediction model across different populations. All statistical tests were two-sided, and *p* < 0.05 was considered statistically significant.

## 3 Results

### 3.1 Differences in demographic characteristics and biochemical indicators between POP and non-POP groups

The numbers of women in POP-Q stages I, II, III, and IV are 14, 54, 35, and 15, respectively. The non-POP group (*n* = 162) included only women with POP-Q stage 0. The POP group had significantly higher age, BMI, waist circumference, parity, and proportion of T2DM compared to the non-POP group. Regarding metabolism-related indicators, FPG, HbA1c, HOMA-IR, TG, and LDL-C were significantly higher in the POP group than in the non-POP group (*p* < 0.05), while HDL-C and 25(OH)D were significantly lower. There were no significant differences in TC and CRP between the two groups. After FDR correction, HOMA-IR, TC, TG, and HDL-C showed marginal significance (Table 1).

**Table 1:** Differences in demographic characteristics and biochemical indicators between the POP and non-POP groups.

Variable	Total (*n* = 280)	None-POP (*n* = 162)	POP (*n* = 118)	*p*-value	FDR
Age	61.11 ± 4.64	60.44 ± 3.91	62.02 ± 5.38	0.007	0.030
Age at menopause	49.89 ± 3.20	49.88 ± 3.04	49.92 ± 3.42	0.923	0.923
BMI	24.8 (23.3–26.8)	24.3 (23.0–26.2)	25.6 (23.5–27.3)	0.005	0.030
Waist circumference	77.92 ± 3.81	77.51 ± 3.64	78.49 ± 3.96	0.036	0.068
Parity				0.019	0.051
<2	104 (37.14%)	70 (43.21%)	34 (28.81%)		
> = 2	176 (62.86%)	92 (56.79%)	84 (71.19%)		
History of hysterectomy	32 (11.43%)	23 (14.2%)	9 (7.63%)	0.129	0.181
Type 2 diabetes mellitus (T2DM)	49 (17.5%)	20 (12.35%)	29 (24.58%)	0.012	0.037
Hypertension	125 (44.64%)	65 (40.12%)	60 (50.85%)	0.097	0.145
Chronic obstructive pulmonary disease (COPD)	16 (5.71%)	8 (4.94%)	8 (6.78%)	0.693	0.809
Chronic kidney disease (CKD)	21 (7.5%)	13 (8.02%)	8 (6.78%)	0.872	0.916
Coronary artery disease (CAD)	39 (13.93%)	18 (11.11%)	21 (17.8%)	0.155	0.204
Osteoporosis	61 (21.79%)	34 (20.99%)	27 (22.88%)	0.816	0.902
Fasting plasma glucose (FPG) (mmol/L)	5.7 (4.0–6.9)	5.4 (3.6–6.6)	6.0 (4.3–7.2)	0.009	0.030
Glycated hemoglobin (HbA1c) (%)	5.6 (4.4–6.4)	5.1 (4.2–6.2)	6.0 (5.0–7.0)	< 0.001	0.001
Homeostasis model assessment of insulin resistance (HOMA-IR)	2.4 (1.2–3.4)	2.1 (1.1–3.1)	2.8 (1.6–3.6)	0.031	0.068
Total cholesterol (TC) (mmol/L)	4.93 ± 0.96	4.82 ± 0.91	5.06 ± 1.03	0.043	0.076
Triglycerides (TG) (mmol/L)	1.7 (1.2–2.3)	1.7 (1.2–2.2)	1.8 (1.3–2.6)	0.034	0.068
High-density lipoprotein cholesterol (HDL-C) (mmol/L)	1.6 (1.1–2.3)	1.7 (1.2–2.2)	1.4 (0.8–2.3)	0.049	0.079
Low-density lipoprotein cholesterol (LDL-C) (mmol/L)	2.82 ± 1.06	2.67 ± 1.01	3.03 ± 1.09	0.006	0.030
C-reactive protein (CRP) (mg/L)	1.8 (0.7–2.8)	1.9 (0.9–2.8)	1.7 (0.6–2.8)	0.380	0.469
25-hydroxyvitamin D [25(OH)D] (ng/mL)	26.7 (21.6–31.2)	27.9 (22.7–32.6)	25.5 (19.8–29.7)	0.004	0.030

### 3.2 Multivariate logistic regression analysis to identify biochemical indicators associated with POP

Multivariable logistic regression analysis showed that each one-standard-deviation increase in FPG (OR = 1.066, 95% CI: 1.009–1.124, *p* = 0.023), HbA1c (OR = 1.117, 95% CI: 1.057–1.180, *p* < 0.001), TG (OR = 1.060, 95% CI: 1.002–1.120, *p* = 0.041), and LDL-C (OR = 1.073, 95% CI: 1.016–1.134, *p* = 0.012) was independently associated with an increased risk of POP. In contrast, each one-standard-deviation increase in 25(OH)D was associated with a reduced risk of POP (OR = 0.934, 95% CI: 0.878–0.985, *p* = 0.010) (Table 2). Collinearity analysis indicated that the variance inflation factors (VIF) of these variables were all less than 2, far below the commonly used threshold of 5, suggesting no multicollinearity among the independent variables (Supplementary Table 1).

**Table 2:** Multivariable logistic regression analysis to identify biochemical indicators associated with POP.

Term	β	*p* value	OR	CI-lower	CI-upper	OR-SD	CI-lower-SD	CI-upper-SD
FPG	0.028	0.023	1.028	1.004	1.052	1.066	1.009	1.124
HbA1c	0.070	0.000	1.073	1.036	1.111	1.117	1.057	1.180
IR	0.017	0.344	1.017	0.982	1.053	1.027	0.972	1.086
TC	0.037	0.209	1.037	0.980	1.098	1.036	0.981	1.094
TG	0.071	0.041	1.074	1.003	1.149	1.060	1.002	1.120
HDL	−0.061	0.052	0.941	0.885	1.000	0.947	0.897	1.000
LDL	0.067	0.012	1.069	1.015	1.126	1.073	1.016	1.134
OHD	−0.010	0.010	0.991	0.983	0.998	0.934	0.878	0.985

FPG: fasting plasma glucose; HbA1c: glycated hemoglobin; IR: insulin resistance; TC: total cholesterol; TG: triglycerides; HDL: high-density lipoprotein cholesterol; LDL: low-density lipoprotein cholesterol; 25(OH)D: 25-hydroxyvitamin D.

### 3.3 Construction of the prediction model

Based on the significant factors and their coefficients identified in section 3.2, a prediction model was constructed as follows:

Prediction Model = FPG (mmol/L) × 0.028 + HbA1c (%) × 0.07 + TG (mmol/L) × 0.071 + LDL (mmol/L) × 0.067–25(OH)D (ng/mL) × 0.01.

**Figure 1:**
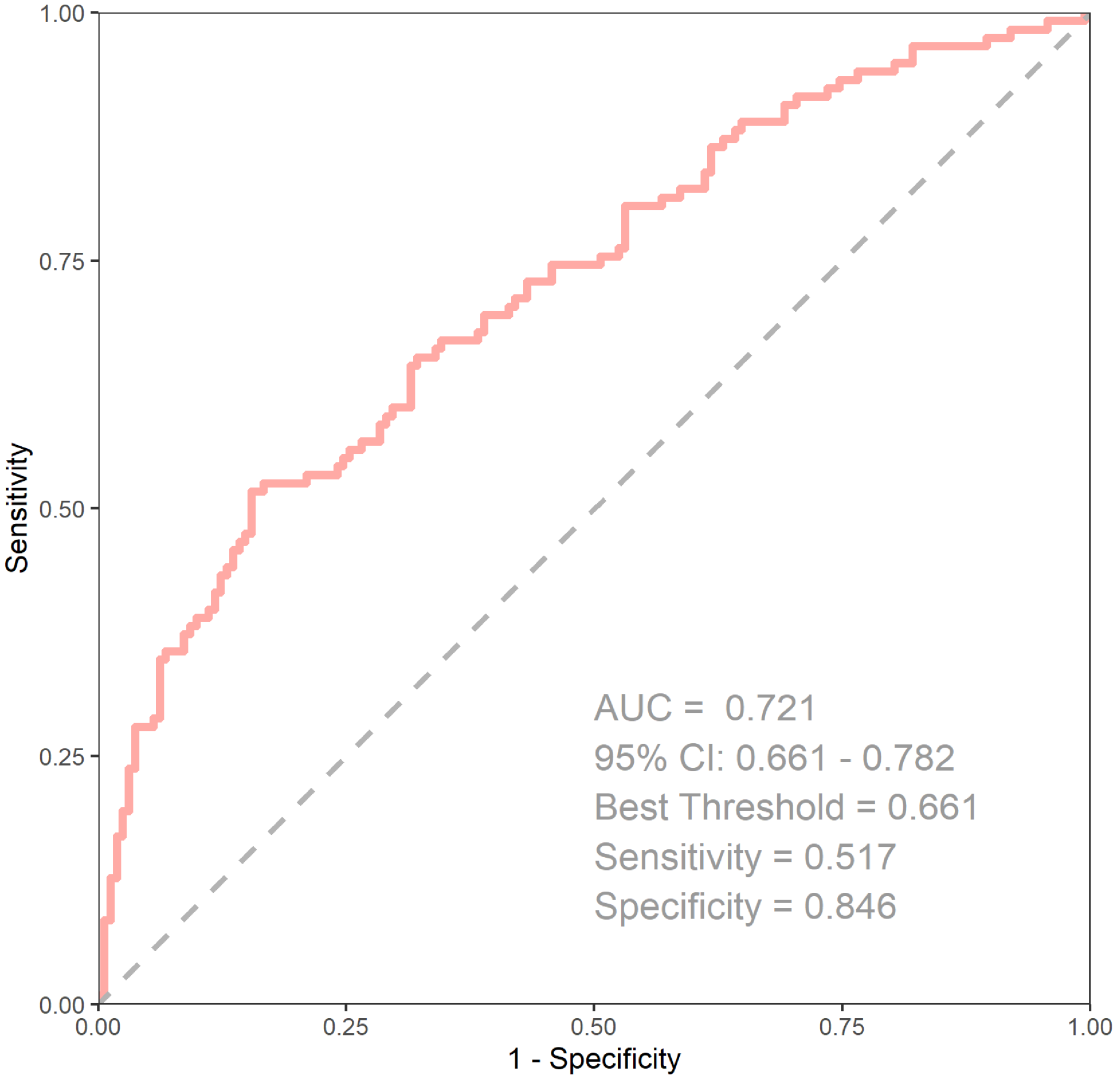
Receiver operating characteristic (ROC) curve of the prediction model.

All variables were used in their original measured values without transformation. After substituting the above parameters of each patient into the equation, a prediction score can be obtained. By comparing the score with the ROC-derived cut-off value (0.661), if the score is higher than the cut-off value, the patient is considered to be at higher risk of POP; otherwise, the patient is classified as low risk. ROC curve analysis showed that the AUC value of this model was 0.721, indicating that the prediction model has moderate discriminative ability (Figure 1). The calibration curve demonstrated good agreement between predicted probabilities and observed outcomes, indicating satisfactory model calibration performance (Supplementary Figure 1).

### 3.4 RCS analysis of the relationship between the prediction model and the risk of POP

**Figure 2:**
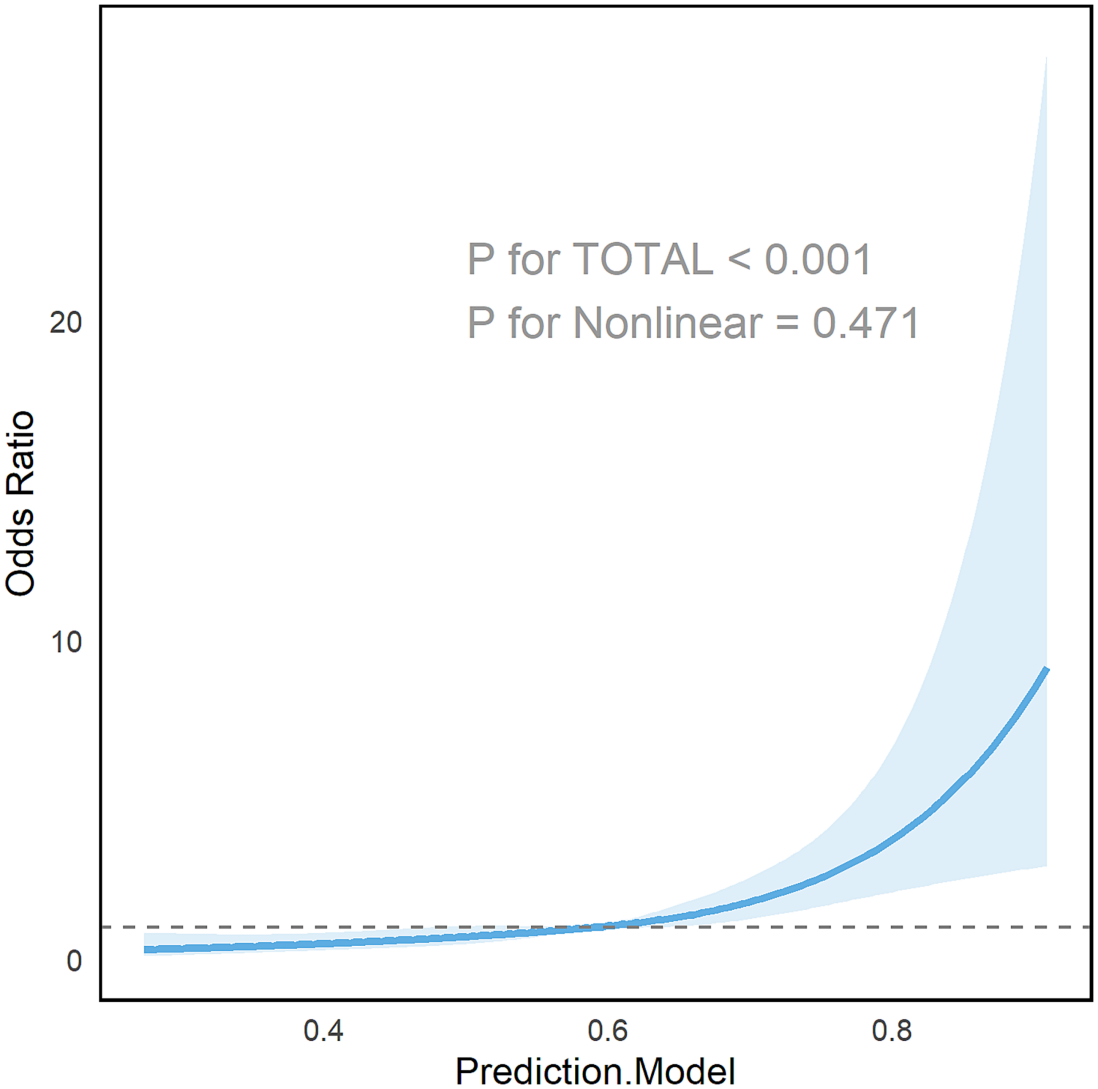
Restricted cubic spline (RCS) analysis of the association between the prediction model and POP risk.

As the prediction model score increased, the risk of POP also increased significantly. The overall association between the two was statistically significant (P for overall < 0.001). The test for nonlinearity showed no significant nonlinear association (P for nonlinear = 0.471), suggesting that the relationship between the prediction model score and the risk of POP follows a linear or approximately linear trend (Figure 2).

### 3.5 Independent predictive effect of the prediction model on POP

**Figure 3:**
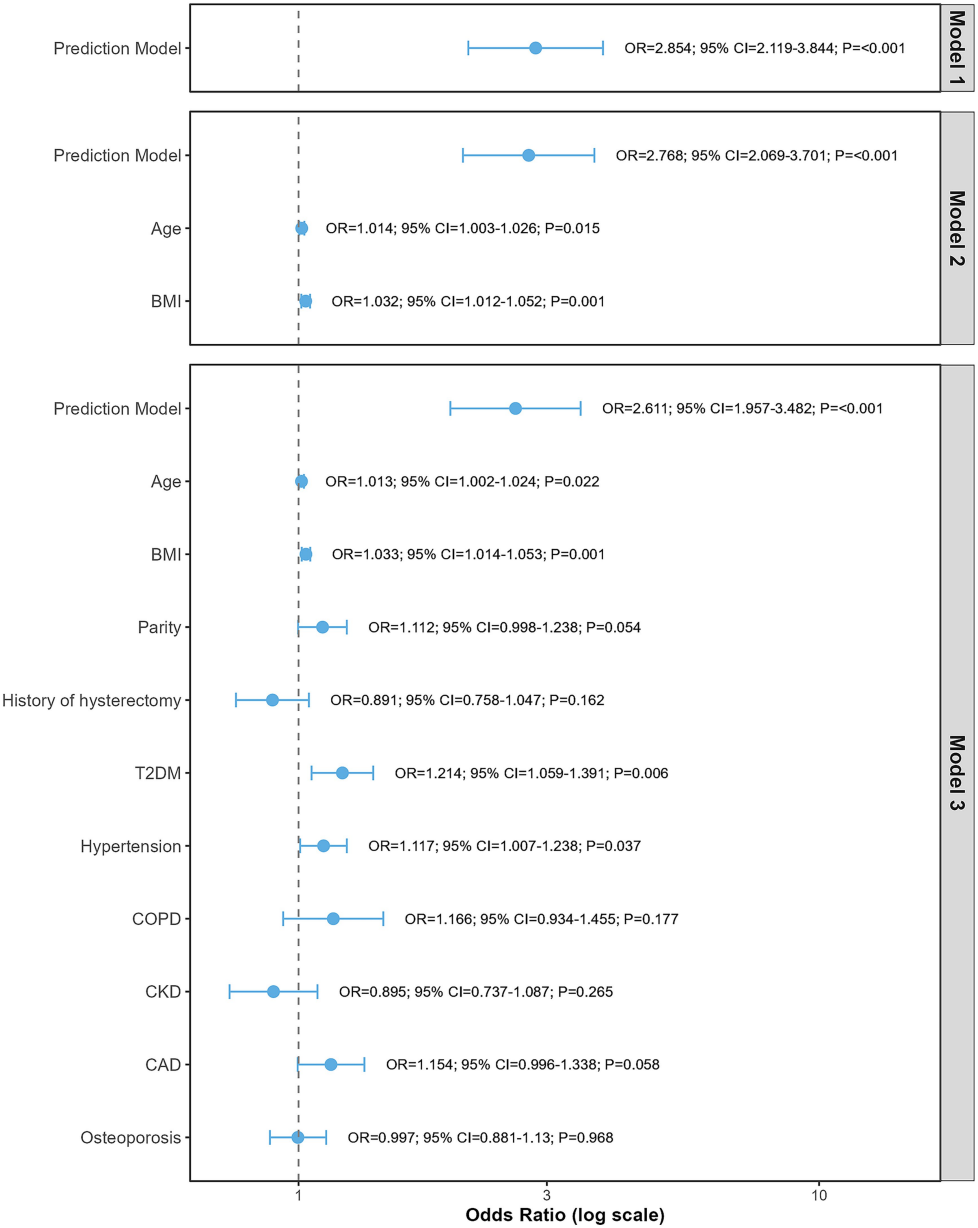
Forest plot of the association between the prediction model and the risk of POP (ORs of 2.854, 2.768, and 2.611 indicate that each 1-unit increase in the prediction score is associated with a 2.854-fold, 2.768-fold, and 2.611-fold increase in the odds of POP).

Across the three differently adjusted models, the prediction model was significantly associated with the risk of POP (all *p* < 0.001). In Model 1, the OR of the prediction model was 2.854 (95% CI: 2.119–3.844). After stepwise adjustment for age, BMI, and multiple comorbidities (Model 2 and Model 3), the effect showed slight changes but remained significant (Model 2: OR = 2.768, 95% CI: 2.069–3.701; Model 3: OR = 2.611, 95% CI: 1.957–3.482). In addition, age, BMI, T2DM, and hypertension were all independent risk factors for POP (*p* < 0.05) (Table 3; Figure 3).

**Table 3:** Independent predictive effect of the prediction model on POP.

	Model 1				Model 2				Model 3			
Term	*p* value	OR	CI-lower	CI-upper	*p* value	OR	CI-lower	CI-upper	*p* value	OR	CI-lower	CI-upper
Prediction Model	< 0.001	2.854	2.119	3.844	< 0.001	2.768	2.069	3.701	< 0.001	2.611	1.957	3.482
Age	-	-	-	-	0.015	1.014	1.003	1.026	0.022	1.013	1.002	1.024
BMI	-	-	-	-	0.001	1.032	1.012	1.052	0.001	1.033	1.014	1.053
Parity	-	-	-	-	-	-	-	-	0.054	1.112	0.998	1.238
History of hysterectomy	-	-	-	-	-	-	-	-	0.162	0.891	0.758	1.047
T2DM	-	-	-	-	-	-	-	-	0.006	1.214	1.059	1.391
Hypertension	-	-	-	-	-	-	-	-	0.037	1.117	1.007	1.238
COPD	-	-	-	-	-	-	-	-	0.177	1.166	0.934	1.455
CKD	-	-	-	-	-	-	-	-	0.265	0.895	0.737	1.087
CAD	-	-	-	-	-	-	-	-	0.058	1.154	0.996	1.338
Osteoporosis	-	-	-	-	-	-	-	-	0.968	0.997	0.881	1.130

BMI: body mass index; T2DM: type 2 diabetes mellitus; COPD: chronic obstructive pulmonary disease; CKD: chronic kidney disease; CAD: coronary artery disease.

### 3.6 Interaction analysis of the prediction model

Among all interaction terms with demographic factors, only the interaction term “prediction model × age” showed a significant association with the risk of POP (*p* = 0.028), and the OR value was greater than 1, indicating that the effect of the prediction model on POP increases with age (Table 4).

**Table 4:** Interaction analysis of the prediction model.

Term	*p* value	OR	CI-lower	CI-upper
Prediction model *Age	0.028	1.048	1.005	1.093
Prediction model *BMI	0.328	0.945	0.843	1.059
Prediction model *Waist circumference	0.190	1.055	0.974	1.144
Prediction model *Parity	0.822	0.928	0.482	1.785
Prediction model *History of hysterectomy	0.797	0.885	0.350	2.239
Prediction model *T2DM	0.464	1.380	0.583	3.270
Prediction model *Hypertension	0.512	1.225	0.669	2.244
Prediction model *COPD	0.468	1.597	0.452	5.636
Prediction model *CKD	0.556	0.698	0.212	2.304
Prediction model *CAD	0.732	0.870	0.392	1.929
Prediction model *Osteoporosis	0.886	1.051	0.536	2.060

BMI: body mass index; T2DM: type 2 diabetes mellitus; COPD: chronic obstructive pulmonary disease; CKD: chronic kidney disease; CAD: coronary artery disease.

### 3.7 Independent predictive effect of the prediction model on the severity of POP

**Figure 4:**
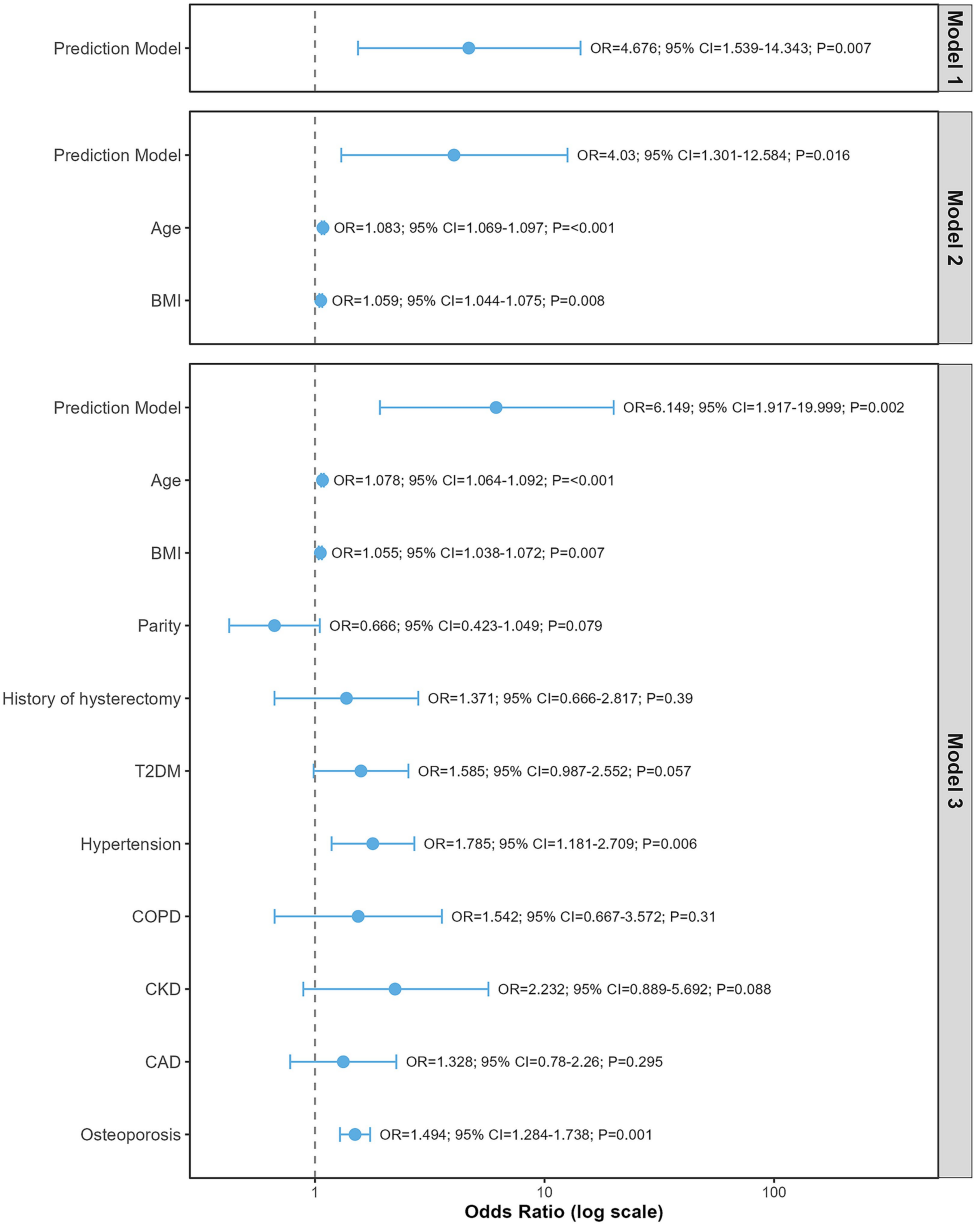
Forest plot of the association between the prediction model and POP severity (ORs of 4.676, 4.030, and 6.149 in Models 1–3 indicate that each 1-unit increase in the prediction score is associated with a 4.676-fold, 4.030-fold, and 6.149-fold increase in the odds of more advanced POP severity).

In the three models adjusted for different factors, the prediction model consistently showed a significant positive correlation with the severity of POP. In Model 1 without adjustment for covariates, the effect of the prediction model was OR = 4.676 (95% CI: 1.539–14.343, *p* = 0.007). In Model 2, after further adjustment for basic variables such as age and BMI, the effect of the prediction model was OR = 4.030 (95% CI: 1.301–12.584, *p* = 0.016). In the fully adjusted final model (Model 3), the effect of the prediction model was OR = 6.149 (95% CI: 1.917–19.999, *p* = 0.002) (Table 5; Figure 4). Bootstrap resampling results showed that the ORs of all models were generally consistent with those of the original regression model (Model 1: OR = 5.665, 95% CI: 1.667–15.517; Model 2: OR = 4.895, 95% CI: 1.240–13.141; Model 3: OR = 8.199, 95% CI: 1.739–23.334) (Supplementary Table 2). All 95% confidence intervals did not cross 1, indicating that the results of the original regression models demonstrated a certain degree of stability.

**Table 5:** Independent predictive effect analysis of the prediction model on the severity of POP.

	Model 1				Model 2				Model 3			
Term	OR	CI-lower	CI-upper	*p*	OR	CI-lower	CI-upper	*p*	OR	CI-lower	CI-upper	*p*
Prediction model	4.676	1.539	14.343	0.007	4.030	1.301	12.584	0.016	6.149	1.917	19.999	0.002
Age	-	-	-	-	1.083	1.069	1.097	< 0.001	1.078	1.064	1.092	< 0.001
BMI	-	-	-	-	1.059	1.044	1.075	< 0.001	1.055	1.038	1.072	< 0.001
Parity	-	-	-	-	-	-	-	-	0.666	0.423	1.049	0.079
History of hysterectomy	-	-	-	-	-	-	-	-	1.371	0.666	2.817	0.390
T2DM	-	-	-	-	-	-	-	-	1.585	0.987	2.552	0.057
Hypertension	-	-	-	-	-	-	-	-	1.785	1.181	2.709	0.006
COPD	-	-	-	-	-	-	-	-	1.542	0.667	3.572	0.310
CKD	-	-	-	-	-	-	-	-	2.232	0.889	5.692	0.088
CAD	-	-	-	-	-	-	-	-	1.328	0.780	2.260	0.295
Osteoporosis	-	-	-	-	-	-	-	-	1.494	1.284	1.738	< 0.001

BMI: body mass index; T2DM: type 2 diabetes mellitus; COPD: chronic obstructive pulmonary disease; CKD: chronic kidney disease; CAD: coronary artery disease.

### 3.8 Sensitivity analysis

Because the simultaneous inclusion of FPG, HbA1c, and T2DM may introduce biological redundancy, sensitivity analyses were conducted according to T2DM status. The results showed that, after excluding T2DM, the findings for both POP risk and POP severity remained largely consistent with the primary analyses, suggesting that the observed associations were robust (Supplementary Table 3).

## 4 Discussion

The results of this study indicate that elevated FPG and HbA1c are significantly associated with the risk of POP in postmenopausal women. The core mechanism may be that collagen is the main structural component of pelvic floor support. A study by Gong and Xia (17) showed that the main collagen subtypes present in the female pelvic floor are collagen type I and collagen type III, where collagen type I affects tissue stiffness and collagen type III is related to tissue elasticity. Reduced collagen synthesis or increased degradation is an important mechanistic basis of POP. As a key metabolic abnormality, hyperglycemia can affect collagen metabolism through multiple pathways, among which the AGEs pathway is one of the most classical and well-studied mechanisms. Hyperglycemia promotes the non-enzymatic glycation of collagen, leading to the formation of advanced glycation end products (AGEs), which play an important role in the pathogenesis of various diseases, such as diabetic complications, rheumatoid arthritis, and osteoporosis (18). AGEs increase collagen cross-linking, reduce elasticity, and weaken tensile strength, thereby causing relaxation of pelvic floor supporting tissues and increasing the risk of POP. Studies have shown that AGEs inhibit the proliferation of human vaginal fibroblasts (HVFs) in patients with POP and reduce the expression of collagen type I through the RAGE and/or p-p38 MAPK and NF-κB–p-p65 signaling pathways (19). In addition, hyperglycemia can stimulate the expression of MMP-1, MMP-2, and MMP-9 (20), leading to imbalance in the regulation of extracellular matrix (ECM) degradation, which also contributes to abnormalities in collagen metabolism.

Previous studies have suggested that triglyceride (TG)-related lipid metabolism disorders may participate in the occurrence and development of POP through mechanisms such as abnormalities in glycerophospholipid metabolic pathways and the reprogramming of fatty acid chain length and saturation (21). Lipid metabolism-related indicators may indirectly promote the accumulation of AGEs, thereby increasing the risk of POP. Under oxidative stress, TG or LDL undergo lipid peroxidation, generating reactive products such as malondialdehyde (MDA) (22), which can cross-link with collagen and promote abnormal extracellular matrix remodeling. During this process, reactive oxygen species (ROS) are also released, and ROS participate in the glycoxidation of glucose, further promoting the formation of AGEs (23), thereby increasing the risk of POP.

Vitamin D may also participate in the occurrence of POP through potential mechanisms. Studies have shown that 25(OH)D inhibits the production and activation of MMP-9 induced by IL-1β, thereby reducing collagen degradation and maintaining extracellular matrix homeostasis (24), thus playing a protective role against POP.

The prediction model constructed in this study has important clinical significance. It integrates multiple indicators including glucose metabolism, lipid metabolism, and vitamin D levels, and can not only be used to assess the risk of POP occurrence but also further predict its severity. The optimal cutoff value obtained from the ROC curve was 0.661, which can preliminarily help clinicians distinguish between high-risk and low-risk populations for POP. This study attempted to transform the traditional structural POP-Q staging based on pelvic physical examination into a non-invasive predictive assessment tool based on blood metabolic indicators. Our model can quantify POP risk in postmenopausal women and may provide Supplementary information for early risk identification, thereby reducing reliance on a single pelvic examination result. However, it should be noted that the model has relatively low sensitivity, and calibration analysis, clinical utility evaluation, and external validation have not yet been performed. Therefore, its clinical applicability remains limited. It is not suitable as a standalone screening tool and should be used in combination with other clinical factors to improve the accuracy of POP risk assessment.

This study has several limitations. As a retrospective study, it may be subject to selection bias to some extent. The study sample was derived from a single center, and unmeasured confounding factors (such as pelvic floor injury history, mode of delivery, and physical activity) may exist, which may limit the generalizability of the findings. In addition, the model has not yet been externally validated, and its stability and generalizability require further evaluation. The confidence intervals of the OR values for predicting POP severity using the model were wider than those for predicting POP occurrence, which may be related to the relatively small sample size and the uneven distribution of POP-Q stages. Future studies with multicenter and larger sample sizes are needed to further validate the stability and generalizability of the model.

## 5 Conclusion

FPG, HbA1c, TG, LDL, and 25(OH)D were independently associated with the occurrence of POP. The prediction model constructed based on these variables demonstrates moderate discriminative ability and shows a linear positive correlation with the risk of POP. Stratified analysis indicates that the association between the prediction model and POP varied across age groups, with a stronger association observed in older participants. Further analysis showed that the prediction model was not only associated with the risk of POP occurrence, but also significantly associated with higher anatomical POP-Q stages. However, the model has not yet undergone internal or external validation. Further validation studies are still required before it can be applied in clinical practice.

## Data Availability

The raw data supporting the conclusions of this article will be made available by the authors, without undue reservation.
