# Supplementary material for: Association between glucose and lipid metabolism-related indicators and the occurrence and severity of pelvic organ prolapse in postmenopausal women
Source: Front Med (Lausanne). 2026 Jul 8;13:1876723. doi: 10.3389/fmed.2026.1876723 (PMC13388566; doi:10.3389/fmed.2026.1876723)
Supplement: Supplementary file 1 [file Supplementary_file_1.docx]

| **Supplementary Table. 1 Multicollinearity analysis of biochemical indicators** | |
| --- | --- |
| **Biochemical indicators** | **Variance Inflation Factor (VIF)** |
| **FPG** | 1.024 |
| **HbA1c** | 1.033 |
| **IR** | 1.058 |
| **TC** | 1.044 |
| **TG** | 1.045 |
| **HDL** | 1.021 |
| **LDL** | 1.046 |
| **OHD** | 1.014 |
| FPG: fasting plasma glucose; HbA1c: glycated hemoglobin; IR: insulin resistance; TC: total cholesterol; TG: triglycerides; HDL: high-density lipoprotein cholesterol; LDL: low-density lipoprotein cholesterol; 25(OH)D: 25-hydroxyvitamin D | |

| **Supplementary Table. 2 Bootstrap resampling analysis for the severity of POP** | | | |
| --- | --- | --- | --- |
| **Model** | **OR** | **CI-lower** | **CI-upper** |
| Model 1 | 5.665 | 1.667 | 15.517 |
| Model 2 | 4.895 | 1.240 | 13.141 |
| Model 3 | 8.199 | 1.739 | 23.334 |

| **Supplementary Table. 3 Independent predictive value of the prediction model for POP and its severity in non-T2DM patients.** | | | | | | | | |
| --- | --- | --- | --- | --- | --- | --- | --- | --- |
| **Variable** | **POP** | | | | **Severity of POP** | | | |
|  | **OR** | **CI-lower** | **CI-upper** | **P** | **OR** | **CI-lower** | **CI-upper** | **P** |
| **Prediction Model** | 2.550 | 1.866 | 3.484 | 0.000 | 4.588 | 1.236 | 17.215 | 0.023 |
| **Age** | 1.015 | 1.003 | 1.027 | 0.017 | 1.051 | 1.011 | 1.092 | 0.020 |
| **BMI** | 1.030 | 1.009 | 1.052 | 0.006 | 0.927 | 0.849 | 1.013 | 0.096 |
| **Parity** | 1.101 | 0.977 | 1.241 | 0.114 | 1.272 | 1.079 | 1.595 | 0.005 |
| **History of hysterectomy** | 0.902 | 0.754 | 1.078 | 0.258 | 1.088 | 0.441 | 2.686 | 0.854 |
| **Hypertension** | 1.123 | 1.002 | 1.259 | 0.047 | 1.977 | 1.220 | 3.223 | 0.006 |
| **CKD** | 0.907 | 0.730 | 1.127 | 0.380 | 3.505 | 1.121 | 11.497 | 0.033 |
| **COPD** | 1.135 | 0.860 | 1.496 | 0.372 | 1.803 | 0.775 | 4.216 | 0.171 |
| **CAD** | 1.186 | 1.009 | 1.393 | 0.040 | 1.513 | 0.833 | 2.755 | 0.174 |
| **Osteoporosis** | 0.997 | 0.864 | 1.151 | 0.970 | 1.261 | 1.017 | 1.686 | 0.045 |
